# Sustained Positivity and Reinfection With SARS-CoV-2 in Children: Does Quarantine/Isolation Period Need Reconsideration in a Pediatric Population?

**DOI:** 10.7759/cureus.12012

**Published:** 2020-12-10

**Authors:** Anjali Patwardhan

**Affiliations:** 1 Child Health, University of Arkansas Health Sciences, Little Rock, USA

**Keywords:** covid-19, sars-cov-2, public health, sustained positivity, quarantine, isolation, transmission

## Abstract

Background: The coronavirus disease 2019 (COVID-19) pandemic is a once in a lifetime public health catastrophe that has driven the world not only into a medical crisis but has pushed to the brink of economic collapse. Prevention of transmission of the replication-competent virus to the susceptible host is the key to the control of COVID-19. The phenomenon of "sustained-positivity," "reinfections," and their role in disease transmission are poorly understood in adults and not even recognized in the pediatric population yet. This information is crucial for ascertaining the quarantine/isolation period for test-positive patients. Most of the time, adult studies' results are extrapolated and applied to children, but severe acute respiratory syndrome coronavirus 2 (SARS-CoV-2) has treated children differently than adults.

Material and methods: The Institutional Review Board (IRB) approval has been obtained. A retrospective electronic chart review of 989 SARS-CoV-2 polymerase chain reaction (PCR)/reverse transcription-PCR (RT-PCR) positive pediatric patients was performed. The aim was to look at the existence of sustained positivity and SARS -CoV-2 reinfection in the pediatric population, as was reported in the adults.

Results: We present our retrospective observational study on 989 SARS-CoV-2 positive pediatric patients; 172 of these had repeated multiple testings, 68 had multiple consecutive positive tests over time, and 27 qualified for sustained-positive status. We also report on four pediatric COVID-19 reinfections.

Conclusion: This is the first report on pediatric SARS-CoV-2 reinfection, one of very few on pediatric SARS-CoV-2 sustained positivity and reinfection. These two phenomena occur in children also as reported in adults but have several differences. The reinfection is possible within one to three weeks of becoming negative as against adults who have been reported to become positive in a minimum of 45-90 days from becoming negative. More extensive reporting is essential to ascertain the accurate quarantine/isolation recommendation in children.

## Introduction

Coronavirus disease 2019 (COVID-19) pandemic is an enormous public health catastrophe in modern history, in the midst of which we are right now. The severe acute respiratory syndrome coronavirus 2 (SARS-CoV-2) is a novel virus and different from most other seasonal viruses, making it difficult to predict its effect on the human host. Most viral infections run a seasonal course, slowing down or disappearing during the summer months, while SARS-CoV-2 has shown a disease spike during summer months. Unlike most seasonal virus infections, SARS-CoV-2 is less likely to affect children and causes less severe diseases except multisystem inflammatory syndrome in children (MIS-C) [1-3]. The viral shedding patterns, variable incubation periods, host susceptibility, "infectious dose," and viral interferences are still not well understood for the SARS-CoV-2. We know that the virus is highly infectious, air-borne, and droplets transmissions occur.

Nevertheless, we do not know if the viral load, duration of exposure, repeat exposure, or reinfections affect the disease's severity [4-5]. In the wake of the uncertainties about the significance of sustained positivity (persistently positive) in COVID-19 patients, a "symptom-based" strategy is considered a better tool than a "test-based" approach by Centers for Disease Control and Prevention (CDC) when deciding on quarantine/isolation measures [6]. Minimal information on the "sustained positivity" of SARS-CoV-2 is available in the pediatric population. No pediatric data on reinfections are available. The patient may be shedding the virus during and after seroconversion, but in humans, the lasting protective value of the antibodies is not yet known [7-8]. We investigated if phenomenon of sustained positivity (persistent secretors) and "re-infection" occurs and its extent in pediatric population.

## Materials and methods

After approval from the Institutional Review Board (IRB) of University of Arkansas Health Sciences & Arkansas Children’s Hospital Systems (USA) IRB #261599, a retrospective electronic medical records (EMR) review was performed to retrieve clinical, testing, and household contacts (HHCs) data on 989 SARS-CoV-2 positive patients. The study aimed to look at if the phenomenon of sustained positivity and SARS -CoV-2 reinfection occurs in the childhood population as it is reported in adults. The SARS-CoV-2 positive patients were identified in EMR by utilizing COVID-19, SARS-CoV-2, PCR/RT-PCR keywords. The patients/subjects included all the children aged 20 years or below who visited the University of Arkansas Medical Sciences, Arkansas Children's Hospital Systems (USA) for their healthcare needs between February 1, 2020 and August 30, 2020. As recommended by CDC, the molecular-genetic tests (PCR/RT-PCR) on nasopharyngeal/nasal swabs were used for the diagnosis. The suspected cases (symptomatic but not tested) were not included in the analysis. The individuals over 20 years of age were defined as adults, and 20 years or below were defined as the pediatric population, for the purpose of this study.

"Strong familial clustering" was defined as three or more COVID-19 patients in a single household. A household disease cluster (HHC) was defined as one to two confirmed cases of SARS-CoV-2 cohabitating in a single home. The cohabitating individuals could be parents, siblings, relatives, and foster families living in the same house. Exposure in institutionalized children while living in dorms or exposed to public places (e.g., hospitalization due to unrelated reasons, hospital deliveries, camping, etc.) was not considered household exposure but was considered community spread. The patients who tested positive but did not have a history of HHCs or known exposure were considered community-acquired and index cases. The incubation period was defined as the period between the exposure and testing positive with or without symptoms. Most studies report that the viral culture from respiratory secretions start becoming negative within the first week from the start of symptoms, and most are negative in 15 days [9-10]. The sustained positivity (persistently positivity) was defined as 17 or more days between two consecutive positive SARS-CoV-2 RT-PCR/PCR positive tests. In our study, the patients continuing to be positive for 17 days or more from their last positive tests were considered sustained positive patients. Certainly, these patients had the "sustained positive period" far longer than that recorded because the exact time of their becoming positive and finally turning negative was not available to register.

The patient demographic characters and symptomatic status of sustained secretors and reinfections are tabulated in tables in the Results section, in detail. Since the number of sustained positive cases and reinfections were small, simple mathematics and percentages were used instead of statistics in data analysis.

## Results

We report 172 pediatric COVID-19 patients who had repeated and consequent SARS-CoV-2 RT-PCR/PCR testing, 68 patients who had two or more consecutive positive tests, and 27 patients who showed sustained positivity. 

Out of 989 COVID-19 test positive patients who were included in this study, 172 patients were identified to have several consecutive repeated tests over months. Sixty-eight COVID-19 patients had two or more positive tests with an average interval between the two subsequent positive tests of 22.5 days (7-110 days), and the median of 14 days. Out of 68 patients, 27 patients qualified for sustained positivity status (40%) as shown in Table 1. The average "positivity period" in these sustained positive patients was 34 days (17-110 days) with a median of 26 days. The repeat testing was performed on these patients for several reasons: social anxiety, repeat exposures, repeated admissions to the hospitals/clinics, and required by the parent's employers/daycare centers. In 172 patients who had repeat testing, four patients turned positive within a short period (one to three weeks) of turning negative (at least once). These patients had repeat testing after repeated exposures or worsening or new onset of symptoms (with no secondary infections or explanation). The more straightforward way to explain the last negative and new positive test in these patients could have been false negative or positive results if they only had a single test performed. But these patients had repeat confirmatory testing, as shown in Tables 1-4. No tests were performed to confirm if patients with reinfection were shedding replication-competent virus. In adult series, the reinfections are recorded at least 45 days from the past infection, but in our study, this period was only one to three weeks [6].

**Table 1:** Sustained-positive patients.

Number of patients with sustained-positive tests	The period (in days) between two consecutive positive tests			Number of tests/patient
	Mean	Median	Range	Mean - 3 times, median - 2 times, range 2-8 times
27	34	26	17-110	

**Table 2:** Demography and characteristics of sustained-positive patients.

Parameters	Observations	Inference in our cohort
Age group	Average age 9.9 years (1.6-18 years)	Occurrence reported in all age groups
Males	5(18.5%)	Females are more likely to secrete the virus for longer periods
Females	22(81.5%)	
Male:Female	0.22:1	
White	5((18%)	The Black children are more likely to shed the virus longer, followed by Hispanic children
Black	14(52%)	
Hispanic	8(30%)	
Asian and Others	0(0%)	
Time of the year the cases presented	June 7(26%), July 7(26%), and August 13 (48%)	Highest incidence in August. Effect of temperature or change in virus strain can be speculated
Strong familial cases (3 or more COVID-19 patients in a single household)	7(26%)	Over one-fourth of sustained-positive patients had strong familial HHC, two-third of patients had HHC, and less than one fourth were Index cases
History of household exposure within one week of presentation	21(78%)	
index cases	6(22%)	
Symptomatic	7(26%)	Sustained secretors are more likely to be asymptomatic
Asymptomatic	20(74%)	
Respiratory symptoms at presentations	6(22%)	
No respiratory symptoms at presentations	21(78%)	Sustained secretors are less likely to present with respiratory symptoms
Fevers	5(18%)	Only a small percentage of sustained shedders are likely to present with fevers
Hospitalization	1(4%)	No sustained shedders had hospitalization due to initial COVID-19, but 4 (15.5%) of these patients had hospital admissions due to non-COVID-19 illnesses during these five months.
Immunocompromised	0(0%)	No sustained shedder was immunocompromised in our cohort. No evidence to suggest that sustained-positive patients had severe disease initially.
Pediatric intensive care unit (PICU) admission	0	
Artificial ventilation	0	

**Table 3:** Demographic and epidemiologic features of re-infected COVID-19 patients.

Patient S. No	Age in years	Sex	Race	Strong familial clustering	Index case	Reason for repeat testing	Time in weeks between last negative to the next positive test
1	1.1	F	Black	Yes	No	Re-exposure	1
2	13	M	Hispanic	No	No	repeated hospital admissions	3
3	0.17	F	White	Yes	No	Re-exposure, recurrence of symptoms	1
4	6	F	Black	Yes	No	Re-exposure, recurrence of symptoms	1

**Table 4:** Diseases characteristics of re-infected COVID-19 patients.

Patient S. No	The severity of initial infection	On immunosuppressants	Symptomatic/asymptomatic during the second infection	Well during interval	Index case	Reason for repeat testing
1	Asymptomatic	No	Asymptomatic	Yes	No	Re-exposure
2	Asymptomatic	Yes	Asymptomatic	Yes	No	Repeated non-COVID hospital admissions
3	Mild	No	Symptomatic	Yes	No	Re-exposure, recurrence of symptoms
4	Mild	No	Symptomatic	Yes	No	Re-exposure, recurrence of symptoms

The black females were more likely to have sustained positivity in our cohort (Tables 24). In our cohort, the patients with strong familial HHCs were more likely to stay sustained positive (Tables 2-4). It cannot be said if the situation of “strong family HHC” is a cause or effect of sustained positivity in these patients. The results showed that interestingly despite being persistent secretors, this group of patients were less likely to be symptomatic (Table 4). The majority of the sustained positive cases were detected in the month of August and their numbers increased from spring to the summer time (Table 2), begging the question if rising environmental temperature can have any effect on sustained positivity. The results also pointed out that majority of symptomatic persistent secretors (from respiratory secretions) presented with nonrespiratory symptoms and without fevers (Table 2).

The reinfected cases had few things in common. Firstly, they had an uneventful interval between their test negativity (after first time testing positive) and retesting positive again. We will call this interval as “X” interval for convenience only. Secondly, all the four reinfected patients were re-tested because they had significant re-exposure to the virus (Table 4). Thirdly, all the reinfected patients were never an index case. Fourthly, the “X” interval in all racial groups in these patients was one week except for Hispanic for which it was three weeks (Table 3). The majority of reinfected cases were females (Table 3), but due to small numbers no conclusive suggestions can be made.

## Discussion

Most studies published so far included symptomatic subjects who tested positive or had suggestive symptoms (suspected cases). Our research has several strengths. This study included only those subjects who objectively tested positive for COVID-19 by molecular genetic testing. Another power of this study is the sizeable pediatric subject population studied over an extended seven-month period. Most likely, the study sample represents the total number of pediatric SARS-CoV-2 cases during the first COVID-19 wave in whole Arkansas state because Arkansas Children's Hospital System is the only dedicated pediatric healthcare system (primary, secondary, and tertiary referral) in the state. Our study covers an extended seven-month period during the evolving pandemic, which includes both the time when travel restrictions/lockdowns were in place and not in place. The study's limitation is that though it is one of the larger pediatric population work-up it is still not enough to get conclusive evidence. It certainly highlights the existence of sustained- positive testing patients and reinfections in the pediatric population and begs the attention and more exploration as in adults. There are very little published pediatric studies to compare our results with right now. Only published study available on children is on 91 pediatric patients from South Korea. The respiratory secretions were positive for the virus for a mean of 17.6 days in 91 children but for a mean of 14.1 days in asymptomatic children [11].

In adults, it is reported that the replication-competent virus load starts reducing by day six or as soon as the patient becomes symptomatic. Still, the patient can continue to stay positive and shed virus in low doses for up to three months, even during/after seroconversion [12-13]. The viral shedding (test positivity) is reported during or after seroconversion in adults, but it is believed, by this time, it does not remain a replication-competent virus [12]. The reported median duration (interquartile range) of viral shedding in respiratory secretions (as measured from the first day of symptoms) in adults is 12-17 days [14-15]. Severe disease (need for artificial ventilation), older age, delay in starting treatment, presence of comorbidities (hypertension without ACE-Inhibitors, COPD, diabetes), use of steroids or immunosuppressives, and male sex were considered high risk factors for sustained shedding in few studies in adult patients [14-19]. In few pediatric COVID-19 reports, the median duration (interquartile range) of viral shedding in respiratory secretions was less in asymptomatic patients (11 days) vs. symptomatic patients (15 days) [20]. Prolonged viral shedding in pediatric COVID-19 patient's feces is reported, and it is suggested that fecal shedding goes on weeks beyond the viral shedding in respiratory secretions [21-22]. The clinical significance of fecal viral shedding from the transmission perspective is not clear yet [22-24]. Sporadic cases of adult COVID-19 reinfections have been reported, and it is suggested that reinfection in adults can occur 45-60 days after recovering from initial COVID-19 [6, 25]. Some researchers have questioned if those were true reinfections.

Observations over a more extended period will inform if SARS-CoV-2 is similar or dissimilar to other seasonal viral pandemics such as the 1918 Pandemic (H1N1 virus), 1957-1958 Pandemic (H2N2 virus), 968 Pandemic (H3N2 virus), and 2009 H1N1 Pandemic (H1N1pdm09 virus). On September 10, 2020, CDC revised its quarantining and isolation recommendations for adults to "symptom-based" from a "test-based" approach for quarantine/isolation and acknowledged that prolonged viral shedding is possible. The current quarantine/isolation recommendations for adults are 10 days for milder and asymptomatic cases and up to 20 days for moderate to severe cases. The immunocompromised adult patients are likely to need extended quarantine/isolation period. The adult quarantine/isolation period begins on the first symptomatic day, and any symptoms and not just respiratory symptoms are considered significant. The day they test positive for the asymptomatic patients is counted as day one for the quarantine/isolation point of view. None of such recommendations are available for pediatric populations as yet. The experience with COVID-19 has shown that it treats adults and children differently; therefore, extrapolating adult data to children is not reasonable. 

On October 19, 2020, CDC revised its quarantine/isolation guidelines for those who get re-exposed to SARS-CoV-2 after recovering from COVID-19 in the past. The current recommendations are not to-quarantine/isolate patients who have recovered from past COVID-19 if re-exposed to the virus [26]. While the countries follow the evolving recommendations of WHO and CDC (similar organizations in other countries), scientists learn more about the significance of reinfections, viral loads, replication-competency of the virus, and sustained positivity. The difficulty is that the symptoms may be due to non-COVID-19 viral syndrome and can still cause false positive alarms in the flu season, considering the symptom-based approach [27]. At this point, only limited information on the occurrence and significance of sustained positivity in adults is available in published literature, but almost none for pediatric patients. Multicenter data over a more extended period are needed to recognize the occurrence and understand the patterns of viral shedding and the significance of sustained positive/persistent positive pediatric patients in the epidemiology of COVID-19. Our study sheds a glimpse of these issues in children, which begs more extensive and prospective trials for confirming the quarantine/isolation periods for children who are not small adults, as COVID-19 has taught us so far. 

## Conclusions

We report that sustained secretors/persistent secretors and reinfections with SARS-CoV-2 also occur in pediatric population as in adults. In the adult population the persistent viral shedding in respiratory secretions is reported up to three months from the first test positivity or appearance of symptoms. We report that in our cohort, the mean duration of virus shedding in respiratory secretion was 34 days (range 17-110). The knowledge of prolonged shedding is crucial when ascertaining the quarantine/isolation periods. The CDC has revised the guidelines for adults in late October 2020, from test-based to symptom-based protocols for quarantine/isolation. For pediatric patients, the current CDC quarantine/isolation guidelines may need re-look in the backdrop of the evolving information on sustained positivity in children. The minimal reported time for reinfection with SARS-CoV-2 in adults is reported between 45 days to 90 days since last negative result. In children, the reinfections occurred in one to three weeks after the last reported negative test. All the four cases of reinfection had few commonalities, they all were never an index case, they were re-tested after turning negative because they had significant re-exposure, they had an uneventful interval period (‘X interval’), the “X” interval in all racial groups in these patients was one week except for Hispanics in which it was three weeks. The majority of reinfected cases were females, and two ever asymptomatic (ever) and two were symptomatic but due to small numbers, no conclusive suggestions can be made at this time.

There is limited to none dedicated data available on sustained positivity and reinfection in children. More extensive and prospective large group studies are needed to conclusively determine if adult guidelines can be extrapolated for children. We know that the SARS-CoV-2 treats children differently.
